# Pharmaceutical pollution alters the cost of bacterial infection and its relationship to pathogen load

**DOI:** 10.1098/rspb.2023.1273

**Published:** 2024-01-10

**Authors:** Lucinda C. Aulsebrook, Bob B. M. Wong, Matthew D. Hall

**Affiliations:** School of Biological Sciences, Monash University, Melbourne, Victoria 3800, Australia

**Keywords:** multi-stressor, fluoxetine, host–parasite interactions, ecotoxicology, virulence, infectious disease

## Abstract

The relationship between pathogen proliferation and the cost of infection experienced by a host drives the ecology and evolution of host–pathogen dynamics. While environmental factors can shape this relationship, there is currently limited knowledge on the consequences of emerging contaminants, such as pharmaceutical pollutants, on the relationship between a pathogen's growth within the host and the damage it causes, termed its virulence. Here, we investigated how exposure to fluoxetine (Prozac), a commonly detected psychoactive pollutant, could alter this key relationship using the water flea *Daphnia magna* and its bacterial pathogen *Pasteuria ramosa* as a model system. Across a variety of fluoxetine concentrations, we found that fluoxetine shaped the damage a pathogen caused, such as the reduction in fecundity or intrinsic growth experienced by infected individuals, but with minimal change in average pathogen spore loads. Instead, fluoxetine modified the relationship between the degree of pathogen proliferation and its virulence, with both the strength of this trade-off and the component of host fitness most affected varying by fluoxetine concentration and host genotype. Our study underscores the potential for pharmaceutical pollution to modify the virulence of an invading pathogen, as well as the fundamental trade-off between host and pathogen fitness, even at the trace amounts increasingly found in natural waterways.

## 1. Introduction

Pathogens are ubiquitous across ecosystems, and the harm they inflict on their hosts has important ramifications for both host and pathogen ecology and evolution [1,2]. The fitness costs that a pathogen inflicts on its host upon infection, such as increased mortality or reduced fecundity, are referred to as the pathogen's virulence [1,3], and is seen as an unavoidable consequence of pathogens exploiting host resources to increase their own transmission success [3,4]. Both host and pathogen evolution, however, can shape this relationship [1,3]. For a pathogen, increased virulence must be balanced against the potential transmission costs of a shorter infectious period or reduced overall proliferation that arises if a host dies too early [3–5]. In turn, hosts can evolve to fight infection by either reducing pathogen burden via limiting pathogen growth (resistance), or by minimizing the damage a pathogen causes (tolerance) [6–8].

This relationship between virulence and the proliferation of a pathogen within a host is integral to theory surrounding disease dynamics [2,9], and has been shown to depend on a variety of biotic and abiotic factors, including host and pathogen genotype [10], the sex and age of the host [11], the transmission mode of the pathogen [12], as well as the environment [13,14]. In particular, the environmental stressors that host organisms confront often reduce their condition, which can result in the expression of higher virulence upon infection [15]. Furthermore, environmental stressors may increase the virulence of a pathogen via reducing the resistance or tolerance of a host, due to the energetic cost involved in coping with these stressors [16,17]. For example, pollutants such as pesticides and heavy metals have been shown to result in increased virulence of various parasites in a range of hosts [13,18–21]. Such increases in virulence are often predicted to result in a reduction in transmission due to earlier host mortality [18], although corresponding changes in pathogen loads are not always found (e.g. [21]). Whether pollutants induce changes in the transmission–virulence relationship via changes in virulence, pathogen replication, or both, is central to understanding the role pollution plays in shaping host–pathogen interactions.

An environmental stressor of increasing concern is pharmaceutical pollution, with hundreds of products being detected globally in rivers, lakes and waterways [22,23]. Following usage, pharmaceuticals are often excreted still in a bioactive form, which leads to the release of these compounds into the environment via wastewater outlets [24]. These contaminants are then slow to degrade in the environment, and can bioaccumulate [25], with concerning implications for exposed wildlife [26], such as feminization of male fish [27–29] and renal failure in vultures [30]. However, little is known about how these pharmaceutical pollutants influence infectious disease traits (see [31,32]), such as the cost of infection or transmission potential, and thus how pharmaceuticals may affect this trade-off, which is fundamental to disease ecology and evolution.

While pollutants such as heavy metals and pesticides frequently decrease host condition or reproductive output [33–35], exposure to pharmaceutical pollutants has sometimes been reported to induce positive effects on growth and reproduction [36,37]. Such effects could potentially result in decreasing the fitness costs inflicted by a pathogen, which could, in turn, have ramifications for pathogen replication. Unlike legacy pollutants (e.g. pesticides, heavy metals), the effects of pharmaceuticals are also frequently non-monotonic, where lower concentrations can result in more severe responses than higher concentrations (e.g. [36,38,39]), perhaps due to these drugs being designed to act in a selective dose-dependent manner [40]. As a result, even trace amounts of pharmaceutical pollutants in the environment have the potential to impact ecosystem dynamics, possibly to a greater degree than areas subject to stronger pollution. These characteristics may result in pharmaceutical pollutants affecting pathogen virulence and transmission in a manner that is quite distinct from more traditional pollutants.

One concerning pharmaceutical contaminant is fluoxetine, marketed as Prozac. Fluoxetine is one of the most commonly detected psychoactive pollutants in the environment [25,41], due to its frequent prescription as an antidepressant [42,43], and slow degradation (half-life of 47–183 days under constant light, and over 10 000 days in darkness [44]). As a selective serotonin reuptake inhibiter, fluoxetine acts to prevent the reuptake of the neurotransmitter serotonin by interacting with the serotonin transporter (5-HTT or SERT), causing the effect of serotonin to be prolonged [45]. Since serotonin and its transporters are evolutionarily conserved in a wide range of taxa, it is unsurprising that exposure to fluoxetine has been found to have effects in a variety of wildlife, including non-monotonic effects on reproduction in invertebrates (e.g. [36,46]) and behaviour in numerous fish species (e.g. [39,47,48]). However, almost nothing is known about how environmental levels of psychoactive pollutants such as fluoxetine could influence the cost of infection to hosts or transmission potential of pathogens (but see [31]), and thus how these contaminants may shape the ecology and evolution of host–parasite dynamics.

In our study, we investigated the consequences of fluoxetine exposure on the cost of infection and pathogen replication, using *Daphnia magna* and its bacterial pathogen *Pasteuria ramosa.* We chronically exposed *D. magna* to two environmentally realistic concentrations of fluoxetine (i.e. nominal concentrations of 30 ng l^−1^ and 300 ng l^−1^), as well as a concentration used for acute toxicology tests (3000 ng l^−1^) and a freshwater control. Within each fluoxetine treatment group, *D. magna* were either exposed to *P. ramosa* spores, or kept as uninfected controls. We then measured the difference in host fitness components, such as fecundity and intrinsic growth [36,49], between infected and uninfected animals in each treatment group, in order to obtain an indication of virulence under each pollution scenario. Additionally, we recorded the spore loads of infected individuals as a measure of pathogen replication, which is commonly used as a proxy for transmission in many study systems [9]. By examining how fluoxetine exposure shifts the relationship between the cost of infection and pathogen replication, we explored whether this fundamental trade-off is sensitive to pharmaceutical pollution. Such information will assist predictions of how pharmaceutical pollution may uniquely shape the evolution and ecology of host–pathogen interactions.

## 2. Methods

### (a) Study system

*Daphnia magna* is a freshwater filter-feeding crustacean that is frequently used as a model in aquatic toxicology (e.g. [50]), as they are sensitive to their environment and provide an essential role in aquatic ecosystems as primary consumers [51]. *Daphnia magna* and the bacterial pathogen *P. ramosa* are also commonly used in disease ecology studies, due to the influence of environmental factors on both host and pathogen fitness (reviewed in [52]). *Daphnia magna* become infected with *P. ramosa* via ingesting spores present in water or sediment [52]. If not cleared in the first few days, the infection is chronic and results in severe reduction in host fecundity, and an increase in body size [52–55]. Upon the death of the *D. magna*, *P. ramosa* spores are released from the cadaver, whereupon they may be ingested by other *D. magna*.

For this study, we used two *D. magna* genotypes derived from single clones: HU-HO-2 (herein HO2) from Hungary and BE-OHZ-M10 (herein M10) from Belgium. These genotypes were chosen for two reasons. First, they are both compatible with the pathogen genotype used in this experiment (C1), and the pathogen is readily able to enter the host and establish an infection [56]. Second, they are known to differ in several life-history traits as well as infection outcomes—for example, M10 tend to have more offspring and larger body sizes than HO2 individuals [36,53], as well as higher pathogen loads in many cases (e.g. [57]). These genotypic differences have frequently been found to give rise to genotype by environment interactions (e.g. [53,57,58]); therefore using these two genotypes will allow insight into whether or not any fluoxetine effects seen are likely to be dependent on the genotype of the animal.

For three generations prior to the experiment, animals from each clone were cultured individually in 70 ml jars, filled with 45 ml of artificial *Daphnia* media [59,60], which was replaced twice a week. Algae (*Scenedesmus* spp*.*) were dispensed into each jar daily according to the growing needs of the animals, from 0.5 million cells per animal on day 1, to 5 million cells per animal from day 8 onwards. All animals were kept in a controlled-temperature room with a constant temperature of 20°C and an 18 : 6 h light–dark cycle. Experimental animals were taken from the third or fourth clutch of parental *D. magna* and maintained under the same standard conditions as parental generations.

### (b) Fluoxetine and pathogen exposure

The experiment was performed in two overlapping time blocks, starting 2 days apart. From day 1 of each block, *D. magna* were exposed chronically to one of the four nominal fluoxetine concentrations: low (30 ng l^−1^), medium (300 ng l^−1^), high (3000 ng l^−1^) and a freshwater control (0 ng l^−1^). The low and medium concentrations are within the range that has previously been detected in the environment, where 30 ng l^−1^ represents levels detected in surface water and 300 ng l^−1^ represents levels found in direct effluent flows from wastewater treatment plants [41]. The high treatment represents a nominal concentration that is less environmentally realistic but represents the magnitude used in acute toxicology studies [61]. These fluoxetine treatments were produced following established protocols [36,39,62,63], by dissolving fluoxetine hydrochloride in a small volume of methanol, and then distributing this methanol into the *Daphnia* media. The control treatment was dosed with the same volume of methanol, but contained no fluoxetine. Fresh dosing of fluoxetine occurred twice weekly at each water change.

Weekly water samples from each fluoxetine treatment were collected after dosing, and sent to Envirolab Services (MPL Laboratories; NATA accreditation: 2901; accredited for compliance with ISO/IEC: 17025) for analysis. Fluoxetine concentrations were derived using gas chromatography–tandem mass spectrometry (7000C Triple Quadrupole GC-MS/MS, Agilent Technologies, Delaware, USA) following methods described in [39]. Measured concentrations were in line with nominal fluoxetine doses (low: 25.87 ± 2.47 ng l^−1^, medium: 197.5 ± 10.3 ng l^−1^ and high: 1900 ± 184 ng l^−1^; see electronic supplementary material), capturing a tenfold difference in concentration between each treatment.

At days 4 and 5, *D. magna* in the disease exposure treatment received 20 000 *P. ramosa* spores. Our study used a full factorial design with 20–36 individuals per treatment (2 *D. magna* genotypes × 2 disease treatments (exposed or unexposed) × 4 fluoxetine treatments). Variation in the number of individuals per treatment was due to differences in infection rate, as well as handling errors. Throughout the experiment, *D. magna* were monitored daily for survival, and dead individuals were frozen in 0.5 ml RO water at −20°C. Offspring were counted at each water change. At 30 days, the body size of all remaining *D. magna* was measured using a scaled binocular microscope and all *D. magna* were frozen individually in 0.5 ml RO water for later inspection of spore production. Intrinsic growth rates (*r*) were calculated using the timing and number of offspring and then solving the Euler–Lotka equation (following [36,64]).

### (c) Spore analysis

Spore counts per animal were measured using an Accuri C6 flow cytometer (BD Bioscience, San Jose, CA, USA) as per [58]. Frozen individuals that had been exposed to the pathogen were thawed and crushed, and 10 μl of each sample was transferred into 190 µl of 5 mM EDTA in a round-bottomed PPE 96-well plate. Each run involved counting 32 wells, which included a total of 12 *D. magna* individuals, each counted twice, as well as 8 wells containing EDTA only as a wash step. Mature spores were identified and quantified based on their distinct size, morphology and fluorescence (in contrast to immature spores, algae or animal debris) using gates based on fluorescence (via the 670 LP filter) and side scatter (cell granularity). Individuals that did not contain mature spores were noted as ‘uninfected’ and excluded from analysis.

### (d) Statistical analysis

Statistical tests were performed using R software version 4.0.3 software (R Development Core Team, 2020). First, we estimated the costs of infection, in regards to the relative changes in fecundity, body size and intrinsic growth, by calculating the difference between each individual's trait value and the mean of the corresponding uninfected animals of the same genotype and fluoxetine treatment. This allowed us an estimate of the loss of fitness experienced by individuals in each treatment group as a result of infection. We then analysed changes in these cost of infection traits (reduction in fecundity, reduction in intrinsic growth, increase in body size) as well as spore loads, via a linear mixed effects model (via lme4 [65]) with fluoxetine treatment, disease exposure, genotype and interactive terms as fixed effect factors, and block as a random effect. Residuals from all fitted models were approximately normally distributed. All *post hoc* tests were generated using the ‘emmeans' package [66] to produce pair-wise comparisons.

We then explored how fluoxetine may also change the relationship between a pathogen's spore load and the fitness costs it imposes on a host, by fitting a series of multiple regression models to the data, akin to an extension of a two-factor analysis of covariance. Each model considered the linear relationships between spore load (the response variable) and the three costs of infection traits (the regression coefficients), with the host and fluoxetine treatments as additional fixed effects and block as a random effect. The series of models tested specific hypotheses regarding how each treatment potentially influenced the partial effects of fluoxetine on spore loads, ranging from a single pattern for all treatments (no interaction terms included, regression coefficients the same for all treatments), to separate patterns for every combination of host genotype and fluoxetine level (including three-way interaction terms, regression coefficients varying in all treatments). Each model containing the terms of interest was compared to a reduced model and the improvement in fit evaluated using a log-likelihood ratio test. Before analysis, in each treatment we rescaled pathogen spore loads to a mean of one (i.e. relative spore loads) and standardized the fitness cost measures (mean = 1, s.d. = 0), following standard selection analysis approaches [67].

## 3. Results

### (a) Fluoxetine exposure did not alter pathogen replication, but shifted the cost of infection

**Figure 1.**
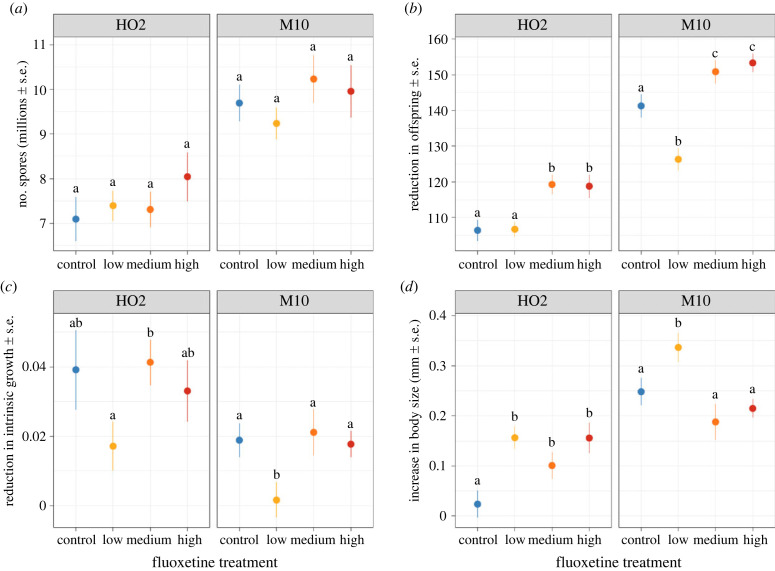
The effect of fluoxetine exposure (control = 0 ng l^−1^, low = 26 ng l^−1^, medium = 198 ng l^−1^, high = 1900 ng l^−1^) on (*a*) mature spore loads, (*b*) reduction in the number of offspring produced, (*c*) reduction in calculated intrinsic growth and (*d*) increase in body size of infected *Daphnia magna* of two genotypes (HO2 and M10). Panels (*b*,*c*,*d*) were calculated as the difference in these traits (offspring, intrinsic growth and body size) from the mean trait value of unaffected animals of each respective fluoxetine treatment group. Points represent treatment means (± s.e.). Lowercase letters indicate significant groupings by *post hoc* comparisons conducted separately for each host genotype (*p* < 0.05).

We found that the number of spores produced per individual did not appear to be significantly influenced by fluoxetine exposure for either host genotype, indicating no change in pathogen replication across a 100-fold change in fluoxetine concentration (figure 1*a* and table 1). There were also no significant differences in survival or infection rate between fluoxetine treatment groups within the 30-day period (see electronic supplementary material). Conversely, the cost of infection, in terms of changes in fecundity, intrinsic growth and body size, appeared to differ between fluoxetine treatment groups (figure 1 and table 1), driven by changes in the trait values of both infected and uninfected (i.e. control) animals across varying fluoxetine concentrations (see electronic supplementary material). The relative decrease in fecundity experienced by an infected individual was dependent on an interaction between fluoxetine treatment and genotype (table 1). For both host genotypes, infected *D. magna* exposed to the medium and high fluoxetine concentrations experienced a greater reduction in the number of offspring produced compared to the control treatment. However, for M10 only, infected *D. magna* exposed to the low fluoxetine concentration had a smaller decrease in offspring than the control (i.e. freshwater) group (figure 1*b*).

**Table 1.** Effects of host genotype, fluoxetine, and their interaction on spore loads, reduction in offspring, reduction in intrinsic growth and increase in body size of infected *Daphnia magna*. Analysis was performed using liner mixed effect models.

trait	term	*χ*^2^	d.f.	*p*-value
spore loads	host genotype	50.752	1	**<0**.**001**
	fluoxetine treatment	3.070	3	0.381
	host genotype × fluoxetine treatment	1.583	3	0.663
reduction in offspring	host genotype	226.463	1	**<0**.**001**
	fluoxetine treatment	66.889	3	**<0**.**001**
	host genotype × fluoxetine treatment	9.980	3	**0**.**019**
reduction in intrinsic growth	host genotype	12.030	1	**<0**.**001**
	fluoxetine treatment	11.506	3	**0**.**009**
	host genotype × fluoxetine treatment	0.209	3	0.976
increase in body size	host genotype	52.095	1	**<0**.**001**
	fluoxetine treatment	22.233	3	**<0**.**001**
	host genotype × fluoxetine treatment	12.371	3	**0**.**006**

In contrast to the reduction in fecundity, infected hosts exposed to the low fluoxetine treatment instead had a significantly smaller reduction in intrinsic growth compared to the other fluoxetine treatment groups (figure 1*c*). This was true for both host genotypes, as we found no interaction between fluoxetine treatment and genotype (figure 1*c* and table 1). Finally, the change in body size was influenced by an interaction between fluoxetine exposure and genotype (table 1). Specifically, for infected HO2 *D. magna*, all fluoxetine treatments resulted in a significantly larger increase in body size compared to controls, whereas for M10, only *D. magna* exposed to the low fluoxetine treatment had a larger increase in body size (figure 1*d*). Despite the variation in responses to fluoxetine exposure between measures of fitness components and host genotypes, in almost all cases, the greatest magnitude of change occurred at the lowest fluoxetine exposure (the one exception being HO2 reduction in fecundity).

### (b) Fluoxetine exposure alters the relationship between pathogen replication and infection cost in a genotype-specific manner

Our above results show that pathogen spore loads remained relatively constant across different fluoxetine levels, but that the fitness costs imposed by the pathogen instead are sensitive to the concentration of fluoxetine that a host encounters. To further explore how fluoxetine exposure influenced the relationship between pathogen replication and cost of infection, we tested how well the changes in fecundity, intrinsic growth, and body size predicted pathogen spore loads (i.e. slopes in a multiple regression), and if these relationships were modified by fluoxetine treatment, host genotype, or a combination of these factors. Our model fitting approach revealed that fluoxetine exposure changes the relationship between pathogen spore loads and the cost of infection for a host. The exact relationship, however, depended on the specific combination of host genotype and fluoxetine concentration, as the model that included all the three-way interaction terms significantly improved the fit compared to the model where relationship varied by host genotype and fluoxetine independently (table 2).

**Table 2.** Candidate analysis of covariance models describing the effects of host genotype and fluoxetine treatment on the relationship between pathogen spore loads and the costs of infection (measured as the reduction in fecundity, increase in body size and reduction in intrinsic growth relative to the trait means of control animals). The models are listed in order of complexity, beginning with a null model where the fitness costs are assumed to be unrelated to pathogen spore loads (model 1), and ending with the most complex model where regression slopes varied for every combination of host and fluoxetine treatments (model 6). A log-likelihood ratio test was used to test if the addition of more complex terms improved the fit of the underlying reduced model.

candidate models	terms added	base model	*χ*^2^	d.f.	*p*-value
1. no slopes	treatment intercepts	—	—	—	—
2. common slopes for all treatments	*Δ*fecundity	1	49.813	3	**<0**.**001**
	*Δ*body size				
	*Δ*intrinsic growth				
3. different slopes for host genotype only	host × *Δ*fecundity	2	8.755	3	**0**.**033**
	host × *Δ*body size				
	host × *Δ*intrinsic growth				
4. different slopes for fluoxetine levels only	fluoxetine × *Δ*fecundity	2	13.373	9	0.146
	fluoxetine × *Δ*body size				
	fluoxetine × *Δ*intrinsic growth				
5. different slopes for host genotype and fluoxetine levels independently	host × *Δ*fecundity	2	22.564	12	**0**.**032**
	host × *Δ*body size				
	host × *Δ*intrinsic growth				
	fluoxetine × *Δ*fecundity				
	fluoxetine × *Δ*body size				
	fluoxetine × *Δ*intrinsic growth				
6. different slopes for every host genotype and fluoxetine levels combination	fluoxetine × host × *Δ*fecundity	5	20.002	9	**0**.**018**
	fluoxetine × host × *Δ*body size				
	fluoxetine × host × *Δ*intrinsic growth				

**Figure 2.**
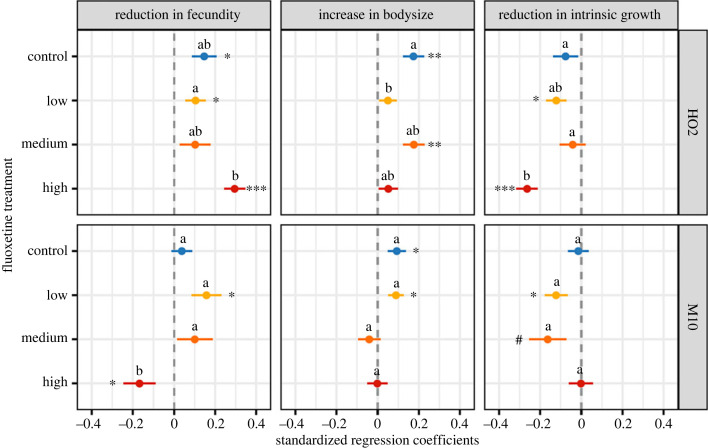
Estimated regression coefficients (± s.e.) for the association between pathogen spore loads and the standardized reduction in fecundity, increase in body size and reduction in intrinsic growth for hosts of two genotypes (HO2 and M10) exposed to one of four fluoxetine treatments (control = 0 ng l^−1^, low = 26 ng l^−1^, medium = 198 ng l^−1^, high = 1900 ng l^−1^). Regression coefficients were estimated separately for each host genotype and fluoxetine treatment combination and spore loads were scaled to a mean of one before analysis. Significance is denoted as * = *p* < 0.05, ** = *p* < 0.01, *** = *p* < 0.001. Marginal significance (*p* < 0.1) is denoted with #. Lowercase letters indicate significant groupings by *post hoc* comparisons conducted separately for each host genotype (*p* < 0.05).

Examining the regression coefficients estimated separately for each treatment combination (figure 2; see electronic supplementary material for partial-residual plots of each regression) revealed that exposure to fluoxetine rarely resulted in a significant change in sign for any of the regression coefficients, regardless of host genotype or fluoxetine concentration. For almost all fluoxetine treatments and host genotypes, spore loads were positively associated with reduction in fecundity and increase in body size, but negatively associated with reduction in intrinsic growth. Instead, fluoxetine exposure modified the strength of these positive and negative relationships in a genotype-specific manner. For HO2 hosts, the high fluoxetine exposure generally resulted in the largest shifts in the slope of the regression, while changes in the low and medium fluoxetine exposure were much milder and often indistinguishable from the control treatment patterns. For M10 hosts, on the other hand, fluoxetine exposure did not appear to result in significant shifts in the regression slopes overall, with the exception of the effect of high fluoxetine exposure on the association between spore loads and reduction in fecundity.

## 4. Discussion

The costs of infection are viewed as an inevitable consequence of a pathogen's replication within its host [4] (reviewed in [9]), but the strength of this relationship has been found to be heavily sensitive to environmental conditions (e.g. [13,14]), including pollutants [13,18,21]. A common assumption of many ecotoxicology studies is that the greater the dose of a pollutant, the larger the negative effect [68]—which has been the case in many studies investigating how conventional pollution affects disease dynamics (e.g. [13,19,69,70]). For many of the fitness costs measured here, however, we instead found that the lowest, rather than the highest, fluoxetine concentration resulted in the largest shifts. For example, exposure to just 30 ng l^−1^ of fluoxetine resulted in infected individuals showing a much milder reduction in intrinsic growth than the control group, while animals exposed to the higher concentrations of fluoxetine experienced the same reduction in intrinsic group as the controls (figure 1*c*). While similar non-monotonic effects have frequently been reported on behaviour and life-history traits (e.g. [36,38,39,48]), and in one case pathogen transmission [31], our results demonstrate that the non-monotonic responses typically induced by pharmaceutical pollutants extend to the reduction in fitness that is caused by a pathogen.

Changes in the costs of infection have previously been reported for other pollutants, such as pesticides [18,71] and heavy metals [19,20], and it is usually assumed that a corresponding shift in pathogen replication, and ultimately transmission, will occur [18]. By contrast, our results show that the influence of fluoxetine pollution is overwhelming felt through the fitness costs imposed by the pathogen alone, as pathogen load remained largely robust to changes in fluoxetine concentration (figure 1*b–d* versus figure 1*a*). At least for pharmaceutical pollutants, shifts in the fitness cost of infection do not necessarily result in an equivalent change in pathogen replication, and thus these pollutants may be altering the fundamental trade-off between virulence and pathogen replication (e.g. figure 2). This could arise if fluoxetine is either acting directly on the way the pathogen is exploiting host resources, or instead modifying the tolerance of the host and its ability to mitigate the damage caused by a pathogen [17,21].

Regardless of the underlying mechanism, pathogens on average produced more spores when there were relatively larger reductions in fecundity and greater increases in body size (figure 2), likely because a greater suppression of reproduction and larger body size result in more resources for a pathogen to exploit [18,72]. Pathogens also produced more spores when the relative reduction in intrinsic growth was smallest (figure 2). The exact mechanism for this relationship remains unclear, but it suggests that while pathogens perform best when total fecundity is reduced, earlier reproduction, on which intrinsic growth heavily depends, may be favourable for pathogens (see [73]). Fluoxetine modified these relationships, however, in a manner that was entirely dependent on the exposure concentration and the genotype of the host (table 2). For some fluoxetine and host genotype combinations, variation in spore loads was most strongly associated with different components of virulence (e.g. medium versus high fluoxetine for host HO2; figure 2). For others, similar fitness costs were linked to spore production, but the relative strengths of each association varied by host genotype or fluoxetine concentration, with some fluoxetine concentrations resulting in little change from the control group, while other concentrations resulted in significant shifts (e.g. variation in the reduction in fecundity regression coefficients across all combinations; figure 2).

Our results indicated that fluoxetine generally did not alter the direction (i.e. sign) of the relationships between spore loads and the different costs of infection, but instead modulated the strength of any association in a genotype specific manner. This transient nature of the relationship between within pathogen replication and the changes in host fitness helps explain why it is has been difficult to detect clear and consistent virulence–transmission trade-offs experimentally [2,9], as other factors, such as pollutants and genotype interactions, can modify which fitness components are most affected as well as the strength of the relationship (e.g. [13,21]). Indeed, our results suggest that pharmaceutical pollution may be a particularly powerful modifier of the relationship between host and pathogen fitness, as the greatest change can arise at the lowest concentration (figure 1), which represents commonly detected levels in natural populations.

Across measures of the cost of infection, as well as its relationship to pathogen load, we have noted that the effect of fluoxetine was frequently dependent on host genotype. Such genotype by environment interactions are frequently reported in disease ecology (e.g. [53,57,58]), as fundamental differences between genotypes make it unlikely for any stressor to affect all genotypes in a uniform manner [74]. The M10 genotype used in our study, for example, has a distinctly larger body size and increased fecundity compared to the HO2 genotype [36,53]. Such differences in life-history traits have the potential to shape the effects of any given stressor, as, for example, larger body size is frequently associated with greater tolerance to pollution [75], as well as higher pathogen loads [76]. Despite this, we did not find evidence that M10 individuals had overall higher tolerance to fluoxetine compared to HO2. Instead, we found that for the M10 genotype, the greatest shifts were largely seen at the lowest fluoxetine concentration, whereas for HO2, higher concentrations of fluoxetine frequently resulted in large shifts. A similar pattern was observed in [36], where low concentrations of fluoxetine induced substantial changes in the life-history traits for M10 *D. magna*, but the same concentration had little effect on HO2 individuals. While the exact mechanism underlying these genotype-specific responses remains to be determined, our study reveals that genotype may be an important factor in determining what concentrations of pharmaceutical pollutants exert the greatest influence on disease dynamics.

Overall, our study demonstrates how pharmaceutical pollution can influence the relationship between host and pathogen fitness by inducing non-monotonic changes in the damage a pathogen causes a host during infection. This result reiterates how testing unrealistically high concentrations of pharmaceuticals, as is common in the field of ecotoxicology (see discussions in [31,36,48]), may distort our understanding of the impact of these emerging pollutants in the wild. It also shows that even ecosystems exposed to trace amounts of pharmaceuticals have the potential to be substantially impacted (see also [31]), highlighting the important role that pharmaceuticals such as fluoxetine may play in shaping the evolution and ecology of host–pathogen interactions in an increasingly polluted world.

## Data Availability

Data are available from the Dryad Digital Repository: https://doi.org/10.5061/dryad.6q573n63h [77]. Electronic supplementary material is available online [78].
